# Human Mesenchymal Stem Cell Response to Lactoferrin-based Composite Coatings

**DOI:** 10.3390/ma12203414

**Published:** 2019-10-18

**Authors:** Madalina Icriverzi, Anca Bonciu, Laurentiu Rusen, Livia Elena Sima, Simona Brajnicov, Anisoara Cimpean, Robert W. Evans, Valentina Dinca, Anca Roseanu

**Affiliations:** 1 Institute of Biochemistry of the Romanian Academy, 060031 Bucharest, Romania; radu_mada@yahoo.co.uk (M.I.); livia_e_sima@yahoo.com (L.E.S.); 2 Department of Biochemistry and Molecular Biology, University of Bucharest, Faculty of Biology, 91–95 Splaiul Independentei, 050095 Bucharest, Romania; anisoara.cimpean@bio.unibuc.ro; 3 National Institute for Laser, Plasma and Radiation Physics, 409 Atomistilor, 077125 Magurele, Romania; anca.bonciu@inflpr.ro (A.B.); laurentiu.rusen@inflpr.ro (L.R.); brajnicov.simona@inflpr.ro (S.B.); 4 Faculty of Physics, University of Bucharest, RO-077125 Magurele, Romania; 5 School of Engineering and Design, Brunel University, London UB8 3PH, UK; robertwevans49@gmail.com

**Keywords:** mesenchymal stem cells, osteogenic differentiation, lactoferrin, polymer composite

## Abstract

The potential of mesenchymal stem cells (MSCs) for implantology and cell-based therapy represents one of the major ongoing research subjects within the last decades. In bone regeneration applications, the various environmental factors including bioactive compounds such as growth factors, chemicals and physical characteristics of biointerfaces are the key factors in controlling and regulating osteogenic differentiation from MSCs. In our study, we have investigated the influence of Lactoferrin (Lf) and Hydroxyapatite (HA) embedded within a biodegradable PEG-PCL copolymer on the osteogenic fate of MSCs, previous studies revealing an anti-inflammatory potential of the coating and osteogenic differentiation of murine pre-osteoblast cells. The copolymer matrix was obtained by the Matrix Assisted Pulsed Laser Evaporation technique (MAPLE) and the composite layers containing the bioactive compounds (Lf, HA, and Lf-HA) were characterised by Scanning Electron Microscopy and Atomic Force Microscopy. Energy-dispersive X-ray spectroscopy contact angle and surface energy of the analysed coatings were also measured. The characteristics of the composite surfaces were correlated with the viability, proliferation, and morphology of human MSCs (hMSCs) cultured on the developed coatings. All surfaces were found not to exhibit toxicity, as confirmed by the LIVE/DEAD assay. The Lf-HA composite exhibited an increase in osteogenic differentiation of hMSCs, results supported by alkaline phosphatase and mineralisation assays. This is the first report of the capacity of biodegradable composite layers containing Lf to induce osteogenic differentiation from hMSCs, a property revealing its potential for application in bone regeneration.

## 1. Introduction

The behaviour of cells mediated by bioresponsive substrates and interfaces represents an important and ever-growing area in tissue engineering. There is an interest in finding innovative strategies for enhancing the efficacy of the biomedical devices used in the treatment of bone diseases such as osteoporosis.

The approaches used are based on either injecting bioactive compounds (i.e., natural protein) in a direct manner or attaching them to a biomaterial surface. The advantage of specific functionalising and addressing/controlling the locally attached proteins over the injecting approach is related to avoiding a rapid clearance of the interest biocompound from the body and side effects. Lactoferrin (Lf) is a multifunctional protein that, apart from its antimicrobial, anti-inflammatory and anti-tumoral effects [1,2,3,4,5], plays a positive role in modulating osteoblast differentiation and inhibits osteoclastogenesis [6,7,8,9]. Different formulations or constructs containing Lf were designed and tested in vitro and in vivo for bone tissue regeneration enhancement. These included Lf incorporation into the collagen membrane [10], hydrogels [11], nanofiber loading [12], or into microspheres [13], or coupled with compounds [14], in order to improve the effectiveness of biomaterials used in bone regeneration. All these data suggest that the Lf-based coatings could be a useful strategy for controlling aspects related to inflammatory or osteogenic responses.

Hydroxyapatite (HA), which is highly biocompatible, osteoconductive and biodegradable and facilitates binding in a functional way to biomolecules, is already an established material for biomedical devices related to bone tissue engineering [15,16,17]. Recent studies demonstrated an effect of Lf-HA nanocrystals on mesenchymal stem cells (MSCs) and pre-osteoblast cells revealing the improved response of the combination of Lf and HA on osteogenic differentiation and an inhibition of osteoclast activity [18,19].

Our previous work demonstrated the synergetic effect of coupling Lf with either antitumoral drugs or osteogenic HA within a biodegradable polymeric matrix [20,21,22]. The hybrid coating of the PEG-PCL-Me-HA-Lf promoted matrix mineralization and osteogenic differentiation of the murine osteoblast cell model in osteoinductive conditions [21] and exhibited low levels of pro-inflammatory TNF-α, high levels of anti-inflammatory IL-10 cytokine, and increased polarization of human THP-1 macrophage cells towards M2 pro-reparative phenotype [22].

The need to use a biodegradable polymeric matrix arises from the long-term biological assays and use, as well as from the necessity to have a controllable and uniform distribution of the biocompounds as coating on the medical device. The future of implant surfaces lies in the design and development of surfaces that interact in a specific way to promote desired processes and minimise detrimental side effects. Biocompatible PEG-PCL copolymers are widely used as controlled drug delivery systems in different therapies [23] and biomedical applications [24,25]. Promising studies on PEG-PCL copolymer surfaces have demonstrated the ability of these materials to maintain the viability and functionality of human MSCs (hMSCs) and to induce morphology changes adapted to the structure and composition of the copolymer for better interaction with the surface [26,27,28,29].

Matrix Assisted Pulsed Laser Evaporation (MAPLE) is an efficient method to obtain multifunctional single or multiple compound biocoatings-polymers, proteins, graphene, nanoparticles and even larger compounds, such as microspheres, bacteria [20,21,22,30,31,32,33].

We recently demonstrated the use of MAPLE as a single-step method for embedding multiple bioactive factors into a biodegradable synthetic polymeric coating without losing the functionality of proteins or drugs [20,22]. It was shown that by entrapping the osteoconductive factor HA and low quantities of Lf within a biodegradable copolymer matrix, both the inflammatory response and osteoblasts’ response were dictated by the coating composition and characteristics [20,22]. 

Given the potential and importance of hMSCs for implantology and cell-based therapy, an understanding of how the characteristics of biointerfaces can be used in controlling and regulating osteogenic differentiation from MSCs is essential. An assessment of results across our previous work has demonstrated that the characteristics of biomimetic interfaces can be used to instigate a specific tissue response. Therefore, we have investigated in this work the effect of Lf and HA embedded within a biodegradable copolymeric matrix on the osteogenic fate of MSCs.

## 2. Materials and Methods

### 2.1. Materials and Solution for Target Preparation

The materials used to obtain the single component and the lactoferrin-based composite coatings were all purchased from Sigma Aldrich: Lf (L0520 SIGMA, Aldrich, Saint Louis, MO, USA), HA nanoparticle powder (677418 Aldrich, Aldrich, Saint Louis, MO, USA), and Poly (ethylene glycol)-*block*-poly (ε-caprolactone) methyl ether (570303 Aldrich, Saint Louis, MO, USA) (PEG-block-PCL Me-average Mn~5000, PCL average Mn~5000). Double distilled water was used to prepare Lf (2%), and HA nanoparticles (1%). PEG-PCL Me solution (1%) was prepared in chloroform. 

### 2.2. Coatings Deposition

The single element and the composite coatings were obtained by the MAPLE method using a single, double and triple module target system and a “Surelite II” pulsed Nd: YAG laser system (Continuum Company, Pessac, France) (266 nm, 6 ns pulse duration, 10 Hz repetition rate) as previously described [20,21] The modular target consisted of frozen solutions of PEG-block-PCL Me copolymer (Co), Lf, HA. In the case of single-component coatings, the solutions were individually prepared and maintained frozen in a copper container using liquid nitrogen. For the composite layers, a modular target system with one or two Teflon concentric rings, depending on the number of components, was used and the materials were frozen separately. The Teflon rings are removed after freezing to avoid the interaction of the laser beam and the exposed Teflon ring. The laser beam was scanned over the target surface and rotated to prevent overheating and drilling due to the laser irradiation. In this way, the laser beam energy is mainly absorbed by the frozen solvent, leading to the vaporization and transfer of the target molecules towards and onto the glass placed parallel, at a distance of 3 cm from the target in the vacuum chamber.

The Ca/P ratio reported for bone is within 1–2 range, depending on the type of bone, age, etc. For the chosen deposition parameters (450 mJ/cm^2^, 0.01 cm^2^ laser spot size, 120 kpulses for Lf (100 μg) 60 kpulses for PEG-PCL-Me and 60 kpulses for HA (134 μg) and according to EDAX measurements, the calculated Ca/P ratio was within the range of 1.3–1.84. The value is close to that previously reported by us for HA-based composite coatings. [20,22]. PEG-PCL-Me assures a gradual degrading period and the number of pulses for Co was chosen to provide a full coverage and/or an entrapping matrix for HA, LF or both.

### 2.3. Surface Characterization

Atomic Force Microscopy (AFM) (XE 100 AFM, Park Systems company, Suwon, Korea) measurements were performed in non-contact mode. Samples were sputter-coated with gold and observed by the FEI Inspect-S scanning electron microscope at an accelerating voltage between 5 and 20 kV in order to analyse the topography of the samples. The elements analysis of the coatings was conducted by the same Scanning Electron Microscopy (SEM) instrument equipped with energy-dispersive X-ray spectroscopy (EDX) system.

The contact angle measurements were performed by the sessile drop method using an optical measuring system (CAM101, KSV, Biolin Surface, Finland) with deionised water. Three drops of the liquid (9 μL) were examined on each substratum, and the contact angle was measured 3 s after the positioning of the drop. 

Surface free energy (SFE) was determined based on the contact angle measurement of two wetting agents: water and di-iodomethane. This calculation was conducted using the concept of polar and dispersion components using the Owens, Wendt, Rabel, and Kaelble (OWRK) method for calculation [34,35,36].

### 2.4. Cell Culture

Biological studies were performed using human mesenchymal stem cells (hMSCs) obtained as previously described [37]. Bone marrow was harvested from one healthy patient undergoing surgery for the orthopaedic implant procedure, with the approval of the Ethics Committee of the University of Medicine and Pharmacy of Craiova (reference No. 68/11.07.2016). The phenotype of hMSC and their osteogenic capacity are presented in Supplementary data (Figures S1 and S2). Cells were cultured in expansion medium-low glucose (1 g/L D-glucose) DMEM + GlutaMax medium (Dulbecco’s Minimal Essential Medium), supplemented with 10% (*v*/*v*) foetal bovine serum (FBS) and 1% (*v*/*v*) streptomycin/penicillin (all from Gibco) and kept at 37 °C with 5% CO_2_. For osteogenic differentiation, hMSCs were cultured in osteoinductive conditions: α-MEM medium (Biochrom AG) supplemented with 82 μg/mL ascorbic acid, 100 nM dexamethasone (Sigma-Aldrich, Saint Louis, MO, USA), and 10 mM β-glycerophosphate (Calbiochem). The medium was changed twice a week during 28 days of culture.

### 2.5. Cell Proliferation Assay

Before in vitro assays, all samples were sterilised by immersion in 1% Penicillin-Streptomycin solution for 15 min.

Cell viability was assessed using the MTS method (CellTiter 96^®^ Aq_ueous_ Non-Radioactive Cell Proliferation Assay, Promega, Fitchburg, WI, USA). hMSCs were cultured on surfaces in a 24-well plate (Costar flat bottom with lid, tissue culture treated) at a density of 5 × 10^3^ cells/cm^2^ in DMEM medium. After 72 h of incubation, the supernatant was removed and replaced with 360 μL of MTS solution (tetrazolium compound in cell culture media) for each well. The plate was incubated at 37 °C, in a humidified atmosphere of 5% CO_2_ in the dark, for 60 min. After the incubation period, 100 μL of the culture solution was transferred to a 96-well clear bottom plate (Nunc, Thermo Scientific) and optical density at 450 nm was measured by a microplate reader (Mithras LB 940 DLReady, Berthold Technologies GmbH &Co. KG, Wildbad, Germany). The absorbance increases in proportion with cell density.

### 2.6. Viability/Cytotoxicity Cell Imaging Assay

The viability of hMSCs after 72 h of cultivation on the surfaces was evaluated using a LIVE/DEAD^®^ Viability/Cytotoxicity kit (Molecular Probes, Eugene, OR). The cells (5 × 10^3^ cells/cm^2^) were incubated for 30 min. at 37 °C with 10 µM of Calcein AM and 4 µM Ethidium homodimer-1 mixture in DMEM medium and then fixed with 4% paraformaldehyde (PFA) for 15 min. Live-cell control is represented by hMSCs plated on the coverslip. Dead-cell control was obtained by the treatment of MSC cells with 70% ethanol for 5 min. ProLong Gold antifade reagent was used to mount the samples which were immediately examined with a Zeiss Axiocam ERc5s with ApoTome.2 slider module using 10× lens, equipped with AxioCam MRm, camera. The images were captured with the AxioVision Rel 4.8 program

### 2.7. Microscopic Evaluation of Cell Adhesion and Morphology

The effect of coatings on the adhesion and morphology of hMSCs was evaluated by fluorescence microscopy. Cells adhered on different surfaces were fixed with 4% p-formaldehyde for 15 min and permeabilised for 3 min at room temperature with 0.2% Triton-X-100. The samples were blocked for 1 h in 0.5% BSA in Phosphate-Buffered Saline (PBS) and then incubated with Alexa Fluor488-conjugated phalloidin (green) for actin filaments detection (1:50, A 12379 Invitrogen, Thermo Fisher Sci., CA, USA). Cell nuclei were labelled for 1 min at room temperature with Hoechst (blue, H 21492 Life Technologies, Molecular Probes, Eugene, OR, USA) at a dilution of 1:3000 in PBS. Before image acquisition, the specimens were mounted in ProlongGold Antifade Reagent (P 36934 Molecular Probes, Life Technologies, Eugene, OR, USA). Fluorescence images were acquired using a Zeiss Axiocam ERc5s Apotom microscope with a 20× lens. The images were analysed with the AxioVision Rel. 4.8 software (Zeiss).

For Scanning Electron Microscopy (SEM) studies, hMSCs seeded on analysed surfaces for 72 h, or 2 to 4 weeks with or without osteogenic factors were fixed with 2.5% glutaraldehyde in PBS for 20 min. The specimens were then subjected, as reported in [38], to dehydration and drying with 70%, 90%, and 100% ethanol solutions, for 15 min twice for each concentration and then 2 rounds of 3 min washing with 50% and 75% hexamethyldisilazane (HDMS, in ethanol) and finally with a 100% HDMS solution. The surfaces were air-dried overnight in a chemical Euroclone AURA 2000 M.A.C. fume hood. 

### 2.8. Determination of Alkaline Phosphatase Activity

Alkaline phosphatise (ALP) activity of hMSCs cultured on different surfaces was determined using the Quantitative Alkaline Phosphatase ES Characterization Kit (SCR066 Merck, Millipore, Darmstadt, Germany). Briefly hMSCs at 14 days of incubation, with or without osteoinductive medium, were incubated with p-nitrophenylphosphate (pNPP) substrate for 20 min at room temperature. The method is based on the capacity of the cellular enzyme to hydrolyse p-NPP into phosphate and p-nitrophenol (yellow coloured). After the reaction is stopped, the absorbance of the coloured compound is measured at 405 nm with a spectrophotometer (Mithras, Berthold Technologies, Bad Wildbad, Germany). The amount of p-nitrophenol produced is proportional to that of ALP present within the reaction. The levels of ALP were extrapolated from a standard curve using recombinant ALP provided in the kit and expressed as ng/mL/sample.

### 2.9. Extracellular Matrix Mineralization Assessment

The samples were employed for detection and quantification of mineralisation occurred at 14 and 28 days after culturing hMSCs in the presence and absence of osteogenic factors on different surfaces. The cells were washed gently with PBS then fixed for 20 min with 4% PFA. After washing with distilled water and carefully removing the entire liquid, the samples were incubated with Alizarin Red S solution (ARS) (40 mM, pH 4.1–4.3) at room temperature for 10 min, repeatedly washed with PBS and microscopically visualised using a TissueFAXS imaging system (Tissue Gnostics, Vienna, Austria). For quantification, Alizarin red staining was extracted for 15 min at room temperature using a solution of 20% methanol and 10% acetic acid in water. Subsequently, the liquid was transferred to 96-well plates and the absorbance read at 450 nm using a Mithras LB940 Berthold spectrophotometer. 

### 2.10. Statistical Analysis

Statistical analysis was performed with GraphPad Prism software using one-way ANOVA with Bonferroni’s multiple comparison tests. Triplicate samples were used in all biological assays to ensure the reproducibility of the results. The data are presented as means ± SD (standard deviation). The *p* values < 0.05 were considered to be statistically significant.

## 3. Results and Discussions

### 3.1. Surface Characterisation of Composite Coatings

**Figure 1:**
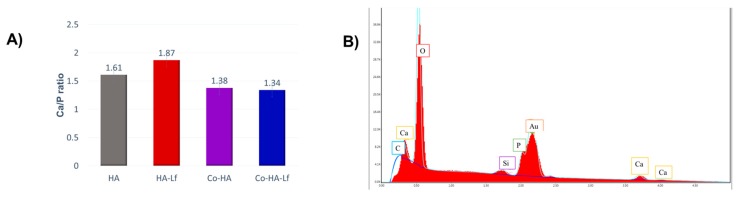
(**A**) Ca/P ratio of composite coatings obtained by Matrix Assisted Pulsed Laser Evaporation technique (MAPLE), (**B**) energy dispersive X-ray (EDX) spectra of hydroxyapatite (HA) coating obtained by MAPLE.

As surface characteristics are strongly correlated to cell response and behaviour, the Lf-based composite coatings characteristics were investigated in order to correlate those properties with the effect on the mesenchymal stem cells (MSCs). The energy-dispersive X-ray spectroscopy data show that the Ca/P ratio in HA containing samples was within 1.3–1.8, which is relatively close to the theoretical value in HA [39,40]. It was reported that HA with Ca/P ratio of 1.67 has good mechanical properties in terms of hardness and fracture toughness which make it a good candidate to serve as a standard for bone tissue regeneration [40]. The data presented in Figure 1A,B show that HA coatings have a Ca/P ratio of 1.61, suggesting a mineral composition very similar to that of bone [39]. The contents of Ca and P in the Lf based coatings were consistently higher than those of the copolymer, with the highest percentages of Ca and P being found for the Lf-HA coatings.

Furthermore, according to previous results obtained by FTIR spectroscopic evaluation, it was demonstrated that the functional groups (characteristic stretching and bending vibrations) of single element coatings, as well as a composite one, are maintained after the MAPLE process at 450 mJ/cm^2^ [20,22].

**Figure 2:**
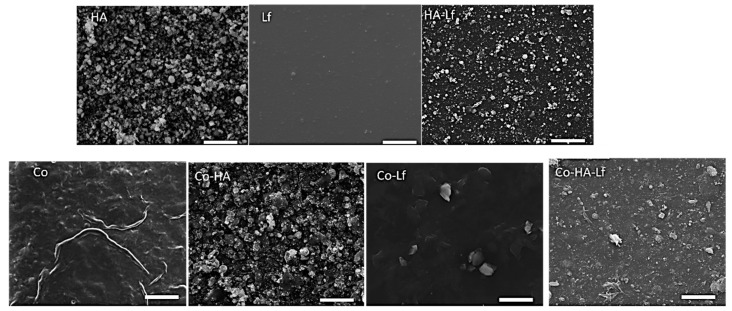
Scanning Electron Microscopy (SEM) images of the top morphology of the coatings obtained by MAPLE. Scale bar: 10 μm.

**Figure 3:**
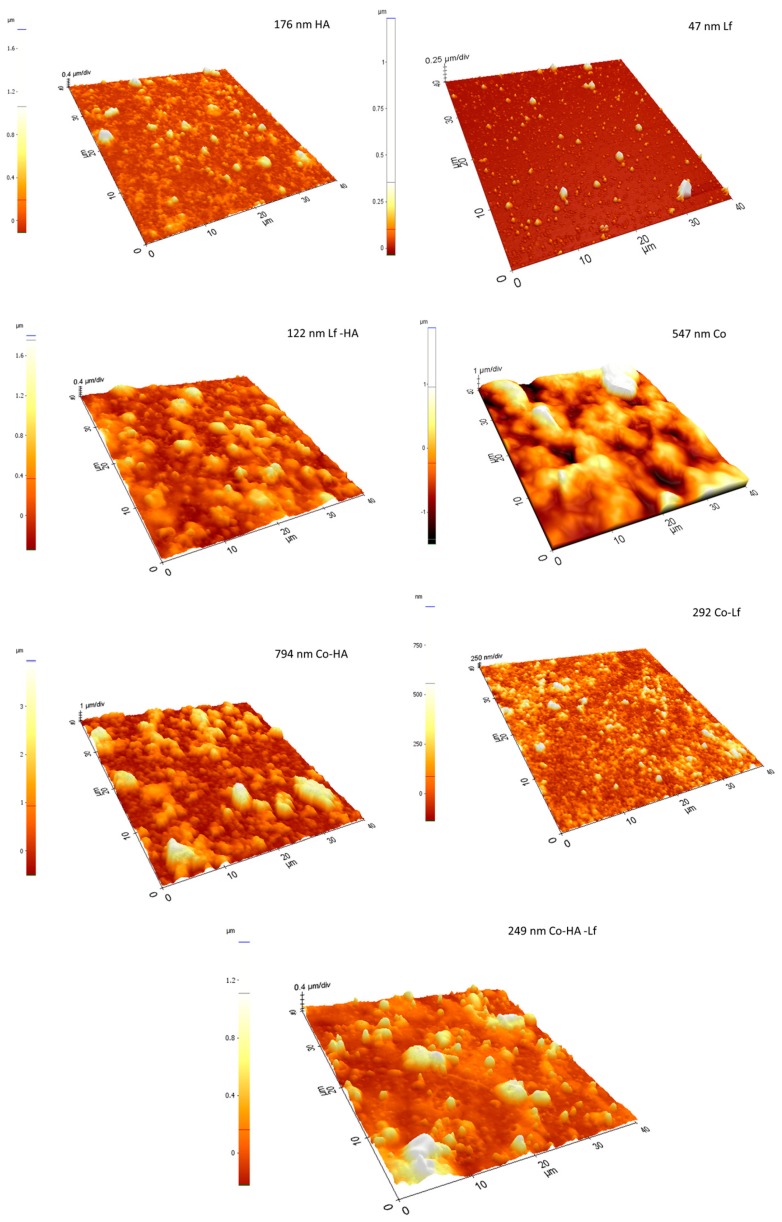
3D Atomic Force Microscopy (AFM) images (40 µm × 40 µm) of single component and composite coatings obtained by MAPLE.

The increase in the number of pulses for each component within the sample’s composition resulted in roughness and hydrophilicity changes as compared to the previously reported samples. These changes in surface morphology and roughness observations are supported by the SEM and AFM results (Figure 2 and Figure 3). For example, in the case of Co, the deposition of a large quantity of material led to its reorganisation on the substrate and wrinkle-like structures that led to an increased surface roughness (794 nm), while the HA, specifically HA-Lf based coatings, were similar to the previous ones [22], being characterised by HA nanoparticle accumulation, without forming cracks on its surface, but with an increased roughness, as shown in Figure 3.

Therefore, the corresponding AFM images also show distinct features for the composite, with the surface root mean square roughness results (obtained from the AFM measurements) revealing increased roughness (47 nm Lf, 794 nm Co HA, 292 Co Lf,122 nm Lf HA, 249 nm Co Lf HA) (Figure 3), indicating clearly that the surface morphology and microstructure change depending on composition. 

**Figure 4:**
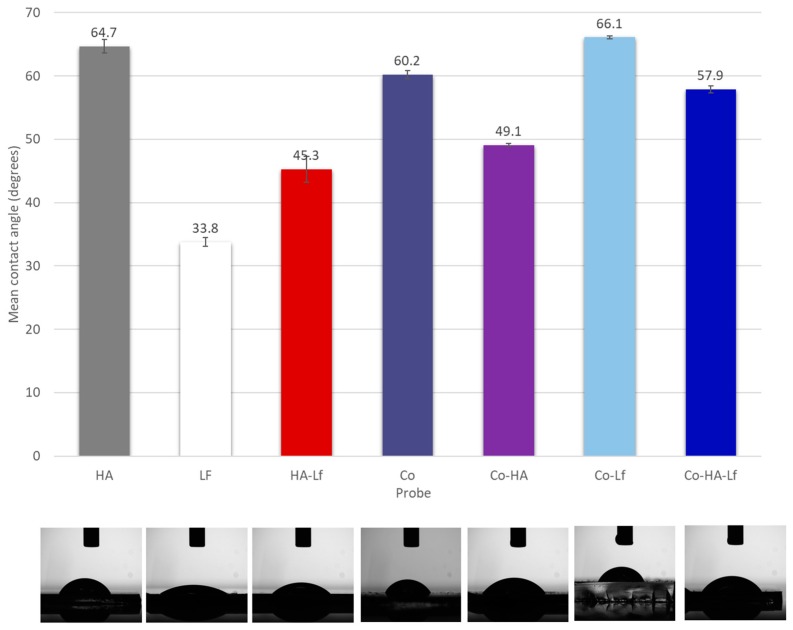
Histogram of contact angle measurements using water as a wetting agent.

The surface charge and hydrophilicity of an implant have been known to influence osteointegration [41,42,43]. When the surface of the biomaterial comes in contact with a biological fluid, the molecules adsorbed create the conditions which will govern cell–surface interactions. One general observation is that protein adsorption is greater on hydrophobic surfaces compared to hydrophilic ones. In our case, the contact angle was significantly lower for Lf, HA-Lf and PEG-PCL-Me-HA (33.83° ± 2.04°, 45.26° ± 0.26° and 49.1° ± 0.65°) than for the HA, PEG-PCL-Me-Lf and PEG-PCL-Me-HA-Lf (64.74° ± 0.71°, 66.07° ± 0.24°, 57.85° ± 0.53°) respectively. A similar contact angle value for HA (62° ± 2°) was obtained by Siniscalco et al. [44]. It was shown by Li et al. that incorporation of PEG improves the hydrophilicity of the multiblock copolymers compared to the PCL homopolymer [45], which was also observed in our case, all of the samples showing hydrophilic character. The protein incorporation into the HA coatings led to a decrease of the contact angle (Figure 4) while its incorporation within the matrix of the copolymer did not induce major changes over the wettability of the composite coatings due to the ability of the PEG-PCL-Me to cover the nanoparticles just like a matrix (Figure 2). The variation in contact angle depending on the type of coatings can be related as well to the surface morphology, specifically roughness. 

Cell shape is strongly correlated with surface properties and generally increases in size with increases in hydrophilicity [46]. On hydrophilic surfaces, cells also show strong focal adhesions formation and stress fibre bundles within 3 h of plating. Therefore, MSCs on materials that permit cell spreading, would tend to adopt an osteogenic phenotype, while those whose spreading is limited would become adipocytes. Conversely, on hydrophobic surfaces, staining for actin is far more diffuse and vinculin staining for focal adhesions is lacking [47]. However, while cell attachment to a biomaterial surface is clearly important for good implant integration, the trend for improved cell behaviour with increasing adhesion is not perfect. Indeed, excessive adhesion may actually be detrimental. One report of high levels of MSC attachment on positively charged surfaces concomitantly showed reduced cell spreading and differentiation [48].

**Figure 5:**
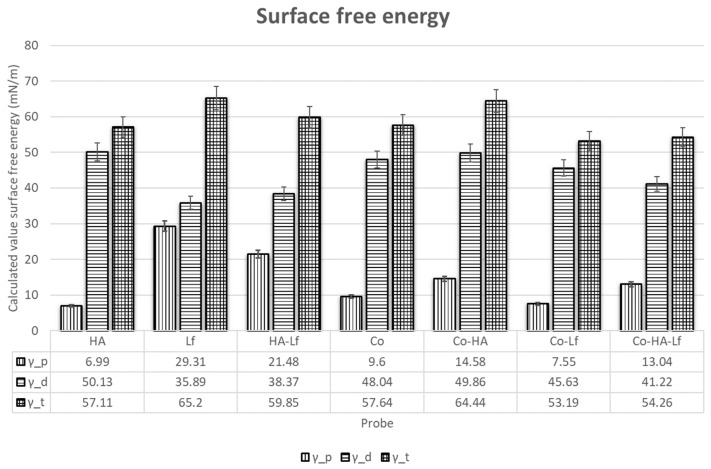
Histogram presenting the total surface free energy, disperse and polar parts.

As previously mentioned, successful orthopaedic implant osteointegration relies on the quick and efficient formation of bone tissue at an implant surface. When biological fluids come in contact with an artificial material, water interactions, protein adsorption, and cell attachment are governed by the surface free energy of the material. Polymers are often considered to have low-energy surfaces due to their covalent and van der Waals bonding, therefore often leading to the surfaces being non-polar, and thus of a hydrophobic nature. As discussed above, cells generally adhere poorly to hydrophobic materials, and thus polymer surface modification is often necessary. In our case, using a laser evaporation technique, we have obtained for the copolymer a surface free energy of 57.64 mN/m with a polar component of 9.6 mN/m. Wei et al. showed that that hydrophilic surfaces strongly supported osteoblast attachment. [49]. The mean values of total surface free energy for HA (57.11 mN/m) is only slightly higher (54.6 mN/m) than the value obtained by Szczes et al. [50]. The total surface energy was equivalent on all the surfaces (Figure 5). The polar component was smaller than the dispersive one for all the coatings. Besides, Lf incorporation induced an increase of the polar component when compared with the original surfaces. Rapid hydration of the layers could facilitate the adhesion of biomolecules [51] and enhance bone apposition in the early healing phase [52,53]. Therefore, the hybrid coating layers with enhanced wettability produced in this study are expected to accelerate osteointegration.

### 3.2. Lf Content in the Polymeric Coating Positively Affects the Cell Proliferation

**Figure 6:**
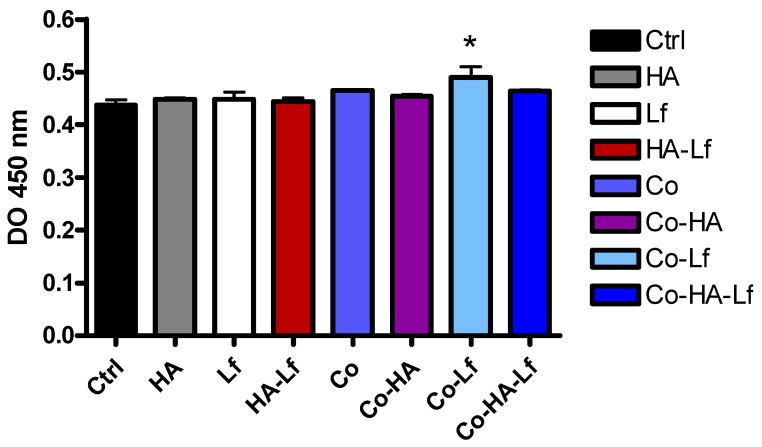
Viability/proliferation of human Mesenchymal stem cells (hMSCs) cultured in direct contact with analysed samples for three days as determined by MTS assay. Data analysis was based on mean ±SD (n = 3). * *p* < 0.05 versus control.

The proliferation of hMSCs was determined after 72 h of culture in direct contact with the surfaces. The MTS assay revealed no toxicity of the surfaces and all samples supported attachment and proliferation of the cells. Cell proliferation after three days of culture was enhanced by addition of human lactoferrin (100 μg/surface) into the polymeric matrix, a significant increase (*p* < 0.05) compared with control being observed (Figure 6). A similar effect was seen on viability and proliferation of MC3T3 murine pre-osteoblast cells cultured on human lactoferrin loaded poly (ε-caprolactone) nanofibres [51]. Lactoferrin was reported to increase proliferation of a mouse pluripotent mesenchymal cell model in a dose-dependent manner [52]. Similar results were reported by Cornish et al. [6] where bovine lactoferrin treatment with concentrations similar to those found in vivo (1–100 μg/mL) on different cell types - rat and human osteoblast-like cells, primary human osteoblast cells, and bone marrow-derived stromal cells-stimulated proliferation. A significant increase in rabbit MSC proliferation was also observed in the case of in vitro Lf treatment for 7 days [18]. In addition, lactoferrin has a protective role against oxidative stress, senescence, and apoptosis of hMSCs, increasing the efficiency of some therapies involving the use of Lf-based biomaterials [53]. Different concentrations of Lf-functionalised biomimetic HA nanocrystals maintained the viability and proliferation of rabbit MSC cells [18] and murine pre-osteoblasts [19] for up to 14 days and Lf and HA in spongy-like hydrogels increased metabolic activity of human adipose-derived stem cells (hASCs) for up to 21 days [54].

Recent studies with PEG-PCL polymeric surfaces have demonstrated biocompatibility of the material and the ability to support normal adhesion, proliferation, and morphology of murine pre-osteoblasts [30]. By incorporating HA and Lf within the polymeric substrate, the proliferation rate of the MC3T3-E1 murine pre-osteoblast line increased from day 1 to day 5. This suggests that the tested surfaces have increased biocompatibility, a high percentage of cells retaining their ability to be metabolically active in direct contact with hybrid substrates [31].

**Figure 7:**
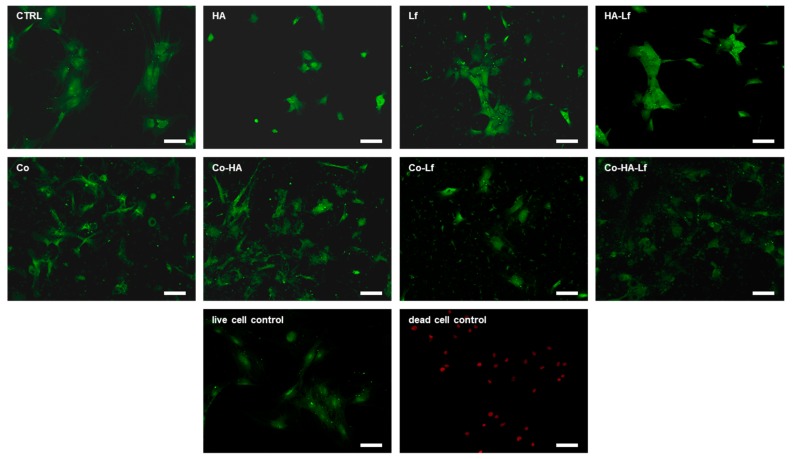
LIVE/DEAD assay. Green-fluorescent live cells and red-fluorescent dead cells are labelled with Calcein AM and ethidium homodimer-1 respectively. Fluorescence microscopy images of hMSCs cultured on different surfaces for 72 h (10×). Scale bar 100 µm.

The proliferation observations are supported by the LIVE/DEAD assay results (Figure 7). No dead cells were observed on either type of surface suggesting that the composition did not impair the adherence and viability of the cells. Fluorescent microscopic inspection showed that all surfaces sustained attachment and proliferation of hMSCs after three days of culture in standard conditions. Different studies showed that Lf alone or in combination with other agents, deposited or incorporated in various supports, sustained the proliferation and viability of mesenchymal cells and bone cells. Also, based on the LIVE/DEAD test, it was demonstrated an increase of up to seven days of the proliferation of murine stromal cells derived from bone marrow encapsulated in a modified recombinant human Lf gel [55] and up to 20 days of MC3T3 murine pre-osteoblasts [56].

### 3.3. Polymeric Coating Control hMSCs Behaviour and Spreading

**Figure 8:**
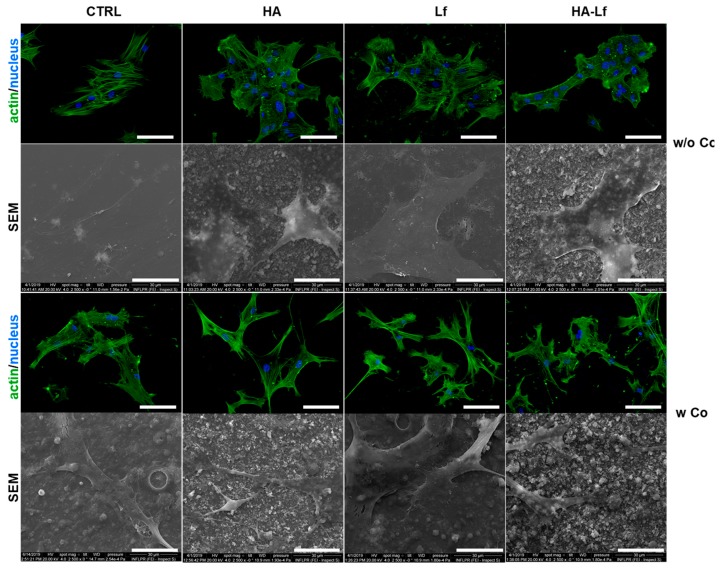
Representative fields of view of human mesenchymal stem cell adhesion and actin cytoskeleton organisation for each surface obtained by fluorescence microscopy. Cell F-actin (green) and nucleus (blue) were examined using a 20× lens. Scale bar is 100 μm. SEM micrographs were taken with 2500× objectives of hMSCs on substrates. Scale bar represents 30 μm.

We subsequently investigated the morphology and adhesion of hMSCs after three days of culture in standard conditions on the different substrates. Immunofluorescent investigation showed that all surfaces allowed monolayer cell attachment and normal morphology development. Phalloidin staining showed a characteristic cytoskeleton organisation, with elongated organised and well-defined actin filaments along the major axis of the cells, on all coatings. However, on surfaces with the copolymeric matrix, hMSCs showed a different behaviour with respect to surface coverage, compared with surfaces without the PEG-PCL component. Surfaces with one component displayed cells with a colony-like proliferation while composite materials seemed to promote the spreading of mesenchymal cells over the surface. Probably due to degradation of the copolymer and exposure of different components, biomaterial surface induced slight modification of the cell morphology with spindle-like extensions anchoring to the surface. Figure 8 shows morphological features and spreading of hMSCs cultured on the analysed surfaces. Additionally, the interaction of hMSCs with the substrate for 72 h was analysed by SEM investigation which confirmed attachment and specific spreading of mesenchymal stem cells on the different substrates. Cells on surfaces without the polymeric component appeared more flat and larger with a greater area and perimeter than hMSCs after 72 h of direct contact with substrates that contain the polymeric matrix. The latter surfaces induced some morphological modification regarding the cell anchorage on the substrates. Cells can be seen branching on the surface with cellular extensions that provide a better interaction with the composite materials. Studies with copolymeric surfaces with different concentrations of PEG-PCL components showed that the composition influences the attachment and spreading/aggregation of mesenchymal stem cells [26]. It seems that cell behaviour on these surfaces is influenced by the length and molar percentage of the polymeric units and by the characteristics of the components with cell-adhesive or cell-repellent effect with an important impact on cell-substrate and cell-cell interactions [27]. Observations regarding the ability to adjust the attachment and aggregation of human MSCs according to PEG-PCL composition were also made by Visan et al. [29]. It was also demonstrated that PEG-PCL copolymer films have the potential to reduce the senescence of hMSCs and maintain stem cell functionality [28]. Cell morphology is closely correlated with stem cell differentiation, and a branched morphology has been reported to be compatible with osteogenic differentiation. Kumar et al. consider that a branched form of cells can act as an osteocyte-like morphology to induce osteogenesis of human bone marrow stem cells in the absence of osteoinduction factors [57].

### 3.4. Alkaline Phosphatase Activity in hMSCs Grown in Contact with Hybrid Surfaces

**Figure 9:**
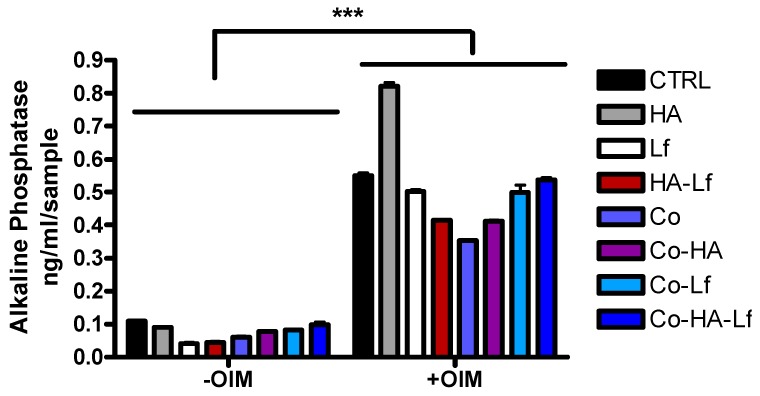
Differentiation of hMSCs to osteoblasts on different surfaces. Quantification of ALP activity at 14 days of hMSCs cultivation with and without osteogenic factors. Data analysis was based on mean ± SD (n = 3). The significance level between groups was *** *p* < 0.001.

To investigate the effect of the surfaces on osteogenic differentiation of hMSCs, ALP activity, an early marker of bone formation was evaluated. The enzyme is expressed in many types of cells but its activity is increased in bone cells, having an important role in mineralisation [58]. The enzyme activity was quantified in the presence or absence of osteoinduction factors after two weeks of the culture of osteoprogenitor cells in direct contact with the surfaces. As shown in Figure 9, after 14 days of culture, ALP activity is statistically increased in osteoinductive conditions compared to standard conditions. Higher values of ALP compared to control (glass) are recorded for cells grown on HA-coated surfaces in osteoinductive conditions. Co-HA-Lf biomimetic polymeric interface exhibit only a slight increase in enzymatic activity compared to control, but higher compared to components alone.

Since Lf treatment led to increased ALP activity, it has been speculated that Lf has the ability to direct the development of mesenchymal stem cells [59] or immature osteoblasts to differentiated phenotypes [60]. Lf treatment also resulted in increased ALP activity in MC3T3-E1 cells, primary osteoblasts [61], but also in human osteosarcoma-derived MG63 cells [60]. Different structures that incorporate Lf are reported in the literature: structures that have been shown to increase the expression level or ALP activity [62,63,64,65]. Lf-functionalised HA nanoparticles [19] or Lf-coated HA nanoparticles [66] led to increased ALP activity in murine pre-osteoblasts (MC3T3-E1) or stem cells derived from rabbit adipose tissue, demonstrating the synergistic effect of these compounds in inducing osteogenic differentiation.

### 3.5. Evaluation of Extracellular Matrix Mineralization

**Figure 10:**
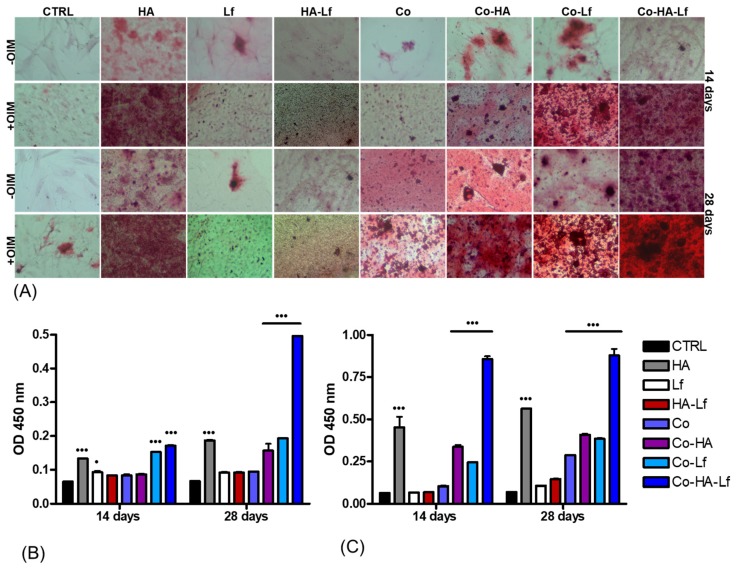
Alizarin Red labelling (**A**) of calcium deposits produced by hMSCs grown on different surfaces. Undifferentiated MSCs with no extracellular calcium deposits appear uncoloured or poorly coloured. The intense orange-red coloration represents the mineralised matrix. Colorimetric quantification of calcium production by hMSCs after 14 and 28 days of culture on analysed samples, in the absence (**B**), and in the presence of osteoinduction factors (**C**) Statistically significant values p < 0.05 and *p* < 0.001 vs control.

Mineralisation is an important late indicator of osteoblastic differentiation as well as an indicator of successful in vitro bone formation [67]. The potential of hybrid coatings to induce osteogenic differentiation of MSC was determined by evaluating mineralisation of the extracellular matrix. The ability of hMSCs to produce calcified matrix was analysed by staining with Alizarin Red S (ARS) solution. The extracellular calcium deposits (Figure 10A) and optical density values of staining solution after 14 and 28 days in the absence (Figure 10B) and respectively in the presence of osteoinduction factors (Figure 10C) were measured. It can be observed that the higher level of mineralisation, characterised by the presence of bone nodules, was detected in the case of hMSCs grown on substrates with bioactive HA and Lf components embedded in polymeric matrix for both short and long periods of time in the presence or absence of osteoinductive factors (Figure 10A).

All analysed surfaces have the ability to induce osteogenic differentiation of hMSCs but with different capacities depending on surface structure. Thus, in standard culture conditions (Figure 10B) low mineral deposition is observed for the surfaces studied, except those covered with HA, Co-HA, Co-Lf, and Co-HA-Lf. The highest level of mineralisation with a significant increase compared with the control (*p* < 0.001) was observed for both 14 and 28 days of culture of hMSCs on Co-HA-Lf surfaces.

The addition of osteoinductive factors led to an increase in mineralisation for all surfaces compared to control at both time points. Significant increases of calcium deposits (*p* < 0.001) were determined (Figure 10C) in the case of Co surface for 28 days and for HA, Co-HA, Co-Lf and Co-HA-Lf coated surfaces for both 14 and 28 days in osteogenic conditions.

The HA-Lf-PEG-PCL surface induces a much higher matrix calcification than any other coating, both at 14 days and 28 days in the presence or absence of osteogenic factors. Such behaviour seems to indicate a possible synergistic effect of HA and Lf released from the polymeric substrate in promoting calcium deposition. An argument in favour of this assumption is the role in osteogenic differentiation and the support of mineralisation described in the literature for Lf, alone [6,61] or incorporated into different structures [56,63,64]. Furthermore, the combination of HA and Lf nanocrystals [18,19,66] or deposited in a polymeric matrix [21] led to increased mineralisation. The differentiation of hMSCs on this type of material could be also correlated with surface characteristics such as wettability, roughness, and surface free energy [67].

**Figure 11:**
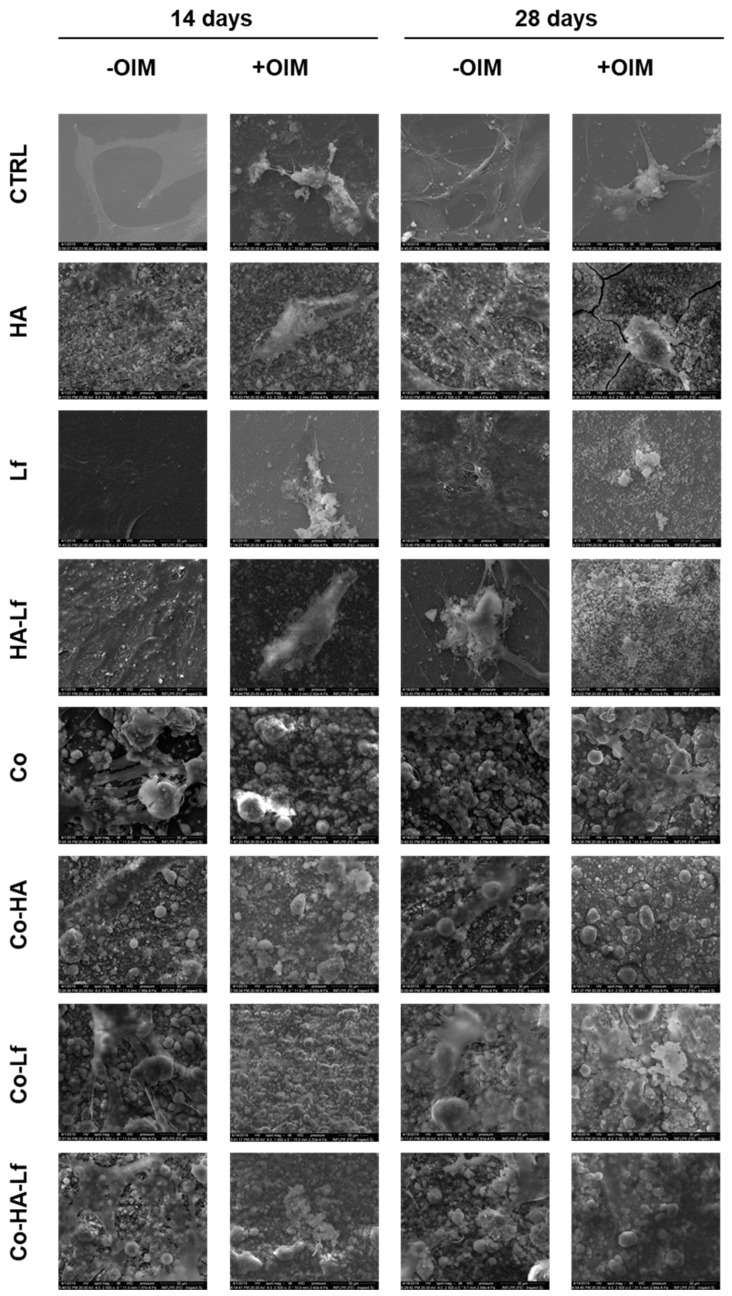
SEM images of hMSCs on each surface, after 14- and 28-days incubation in the presence and absence of the osteoinduction medium (magnification order 2500×—scale bar 30 μm).

Our results are in agreement with that reported in the literature regarding the moderate hydrophilic character of surfaces for osteogenic differentiation. A water contact angle between 50° and 70° seems to favour initial steps of adhesion of pre-osteoblast cells [68] and promote spreading, proliferation and osteogenic differentiation of mouse and human MSCs [69]. Our results are supported by those obtained by SEM microscopy, the images in Figure 11 showing the presence of mineralization nodules on analysed surfaces at different time points in the absence or presence of osteoinduction factors.

## 4. Conclusions

In this work, we report the effect of hydrophilic Lf based composites coatings obtained by MAPLE onto MSCs. 

The coatings were characterised by contents of Ca and P in the Lf-based coatings were close to a Ca/P ratio of 1.61, suggesting a mineral composition similar to that of bone. The distinct roughness and morphological features of the composite were shown by AFM and SEM, indicating the change in surface morphology and microstructure depending on composition. Contact angle and surface energy measurements showed that Lf incorporation into the HA coatings led to a decrease of the contact angle while its incorporation within the matrix of the copolymer did not induce major changes over the wettability of the composite coatings. It also induced an increase of the polar component when compared with the original surfaces. 

The biocompatibility assays revealed the absence of a cytotoxic effect of the studied variants. Our results showed that HA and Lf incorporation into the PEG-PCL-Me polymeric layer promoted hMSCs adhesion and positively modulated morphology and cell spreading associated with an increase in the capacity of osteogenic differentiation, cells adapting to the surface characteristics. Co-HA-Lf surface up-modulated ALP activity and has been shown to be most effective in promoting bone regeneration. A significant improvement of the process of extracellular matrix mineralisation, both in osteoinductive conditions and in the absence of osteoinduction factors, was also demonstrated.

The incorporation of HA and Lf into the copolymer matrix proves to be a method of interest for the manufacture of bioactive surfaces with excellent biocompatibility and osteogenic promotion, properties useful for their application in bone regeneration.

## Supplementary Materials

The following are available online at https://www.mdpi.com/1996-1944/12/20/3414/s1, Figure S1: hMSC immunophenotyping by flow cytometry, Figure S2: (A) Alkaline phosphatase enzymatic activity of primary cells grown in osteoblast differentiation media. (a) Dermal fibroblasts, (b) MSC and, (c) MSC P2 differentiated osteoblasts were tested in the NBT-BCIP substrate solution. (B) Osteoblast mineralisation assay. (a) Dermal fibroblasts, (b) MSC and, (c) MSC P2 differentiated osteoblasts were incubated with Alizarin Red, which specifically binds Ca^2+^ ions.
