# Supplementary material for: Human Mesenchymal Stem Cell Response to Lactoferrin-based Composite Coatings
Source: Materials (Basel). 2019 Oct 18;12(20):3414. doi: 10.3390/ma12203414 (PMC6829495; doi:10.3390/ma12203414)
Supplement: Supplementary file 1 [file materials-12-03414-s001.pdf]

materials

MDPI

*Supplementary Material*

# Human Mesenchymal Stem Cell Response to Lactoferrin-based Composite Coatings

**Madalina Icriverzi [1,2], Anca Bonciu [3,4], Laurentiu Rusen [3], Livia Elena Sima [1], Simona Brajnicov [3], Anisoara Cimpean [2], Robert W. Evans [5], Valentina Dinca [3,*] and Anca Roseanu [1,*]**

1 Institute of Biochemistry of the Romanian Academy, Bucharest, Romania; radu_mada@yahoo.co.uk (M.I.); livia_e_sima@yahoo.com (L.E.S.)
2 Department of Biochemistry and Molecular Biology, University of Bucharest, Faculty of Biology, 91–95 Splaiul Independentei, 050095, Bucharest, Romania; anisoara.cimpean@bio.unibuc.ro
3 National Institute for Laser, Plasma and Radiation Physics, 409 Atomistilor, 077125 Magurele, Romania; anca.bonciu@inflpr.ro (A.B.); laurentiu.rusen@inflpr.ro (L.R.); brajnicov.simona@inflpr.ro (S.B.)
4 Faculty of Physics, University of Bucharest, RO-077125 Magurele, Romania;
5 School of Engineering and Design, Brunel University, UB8 3PH London, UK; robertwevans49@gmail.com
* Correspondence: valentina.dinca@inflpr.ro (V.D.); roseanu@biochim.ro (A.R.)

We provide new evidence of in vitro differentiation assays performed to validate hMSC osteogenic potential (Supplementary Figure 2). To determine the capacity of cells to undergo osteogenic differentiation (Supplementary Figure 2) we tested alkaline phosphatase activity at day 14 and mineralization at the end of the differentiation period, specific features of bone cells. Controls were human dermal fibroblasts (provided by Dr. Lucia Moldovan, INCD-SB, Bucharest, Romania) and MSC grown without differentiation factors. The results showed that ALP activity is significantly intensified after osteoblastic differentiation of hMSC, and dermal fibroblasts (negative control), using NBT/BCIP (nitro blue tetrazolium chloride/5-bromo-4-chloro-3-indolyl-phosphate, Roche) staining.

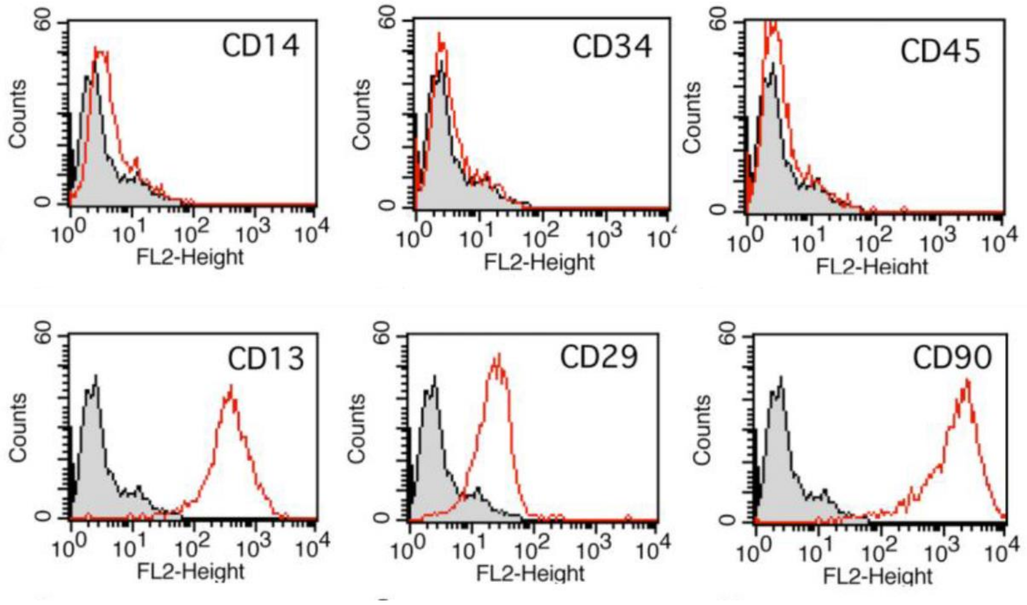


**Figure S1.** hMSC immunophenotyping by flow cytometry.

The phenotype of hMSCs was investigated by flow cytometry method using a FACSCalibur instrument (Becton Dickinson) after labeling the cells with antibodies against specific MSC and HSC (Hematopoietic stem cells) markers (BD Biosciences) (Supplementary Figure 1). Results showed that

cells were negative for HSC and immune cell markers (CD14, CD34, CD45) and positive for MSC markers (CD13, CD29, CD90) (Supplementary Figure 1) thus the immunophenotype of hMSCs was validated at passage two after cell isolation.

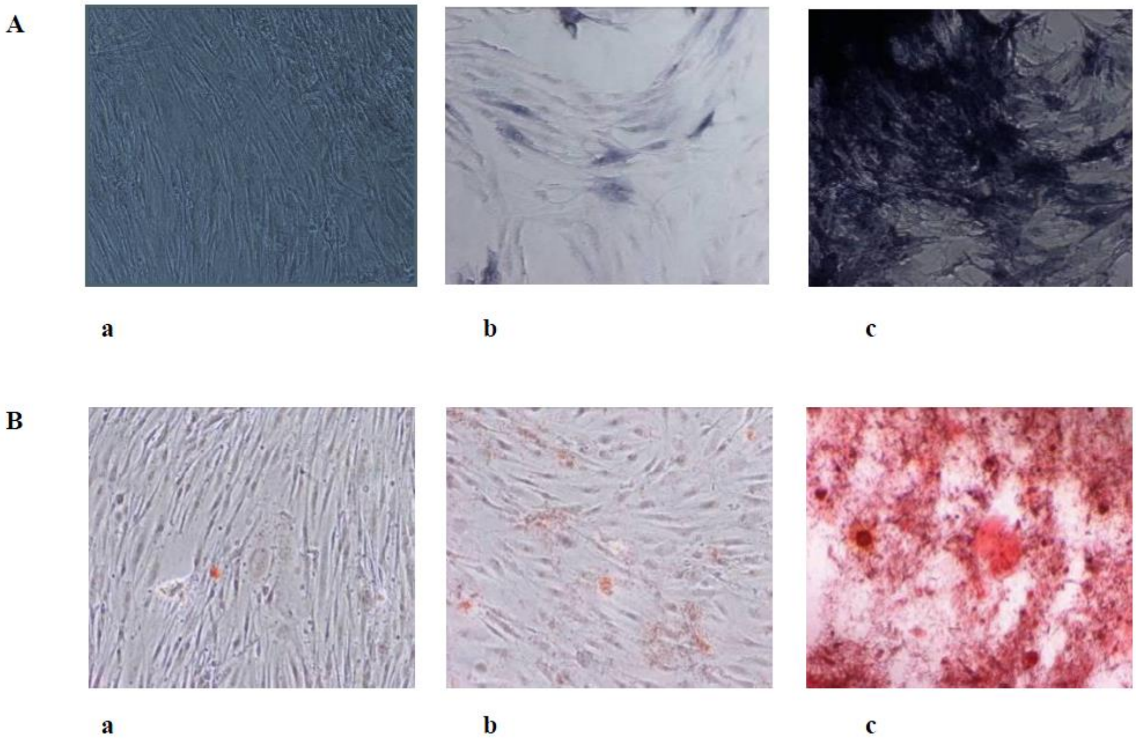


**Figure S2.** (**A**) Alkaline phosphatase enzymatic activity of primary cells grown in osteoblast differentiation media. (**a**) Dermal fibroblasts, (**b**) MSC and, (**c**) MSC P2 differentiated osteoblasts were tested in the NBT-BCIP substrate solution. (**B**) Osteoblast mineralisation assay. (**a**) Dermal fibroblasts, (**b**) MSC and, (**c**) MSC P2 differentiated osteoblasts were incubated with Alizarin Red, which specifically binds $Ca^{2+}$ ions.
